# Inhibitory Effect of Naphthoquinone-Tryptophan Hybrid towards Aggregation of PAP f39 Semen Amyloid

**DOI:** 10.3390/molecules23123279

**Published:** 2018-12-11

**Authors:** Guru KrishnaKumar Viswanathan, Satabdee Mohapatra, Ashim Paul, Elad Arad, Raz Jelinek, Ehud Gazit, Daniel Segal

**Affiliations:** 1 School of Molecular Microbiology & Biotechnology, Tel Aviv University, Tel Aviv 69978, Israel; guru.vgkk@gmail.com (G.K.V.); satabdeemohapatra@gmail.com (S.M.); ashim.ismu@gmail.com (A.P.); ehudg@post.tau.ac.il (E.G.); 2 Department of Chemistry, Ilse Katz Institute (IKI) for Nanoscale Science and Technology, Ben Gurion University of the Negev, Beer Sheva 84105, Israel; eladarad.ea@gmail.com (E.A.); razj@bgu.ac.il (R.J.); 3 Sagol Interdisciplinary School of Neurosciences, Tel Aviv University, Tel Aviv 69978, Israel

**Keywords:** polypeptide aggregation, amyloid inhibition, NQTrp, PAP_248–286_ peptide, semen amyloids

## Abstract

PAP_248–286_, a 39 amino acid peptide fragment, derived from the prostatic acid phosphatase secreted in human semen, forms amyloid fibrils and facilitates the attachment of retroviruses to host cells that results in the enhancement of viral infection. Therefore, the inhibition of amyloid formation by PAP_248–286_ (termed PAP f39) may likely reduce HIV transmission in AIDS. In this study, we show that the naphthoquinone tryptophan (NQTrp) hybrid molecule significantly inhibited PAP f39 aggregation in vitro in a dose-dependent manner as observed from the ThT assay, ANS assay, and transmission electron microscopy imaging. We found that even at a sub-molar concentration of 20:1 [PAP f39:NQTrp], NQTrp could reduce >50% amyloid formation. NQTrp inhibition of PAP f39 aggregation resulted in non-toxic intermediate species as determined by the vesicle leakage assay. Isothermal titration calorimetry and molecular docking revealed that the binding of NQTrp and PAP f39 is spontaneous, and NQTrp predominantly interacts with the polar and charged residues of the peptide by forming hydrogen bonds and hydrophobic contacts with a strong binding energy. Collectively, these findings indicate that NQTrp holds significant potential as a small molecule inhibitor of semen amyloids.

## 1. Introduction

**Figure 1:**
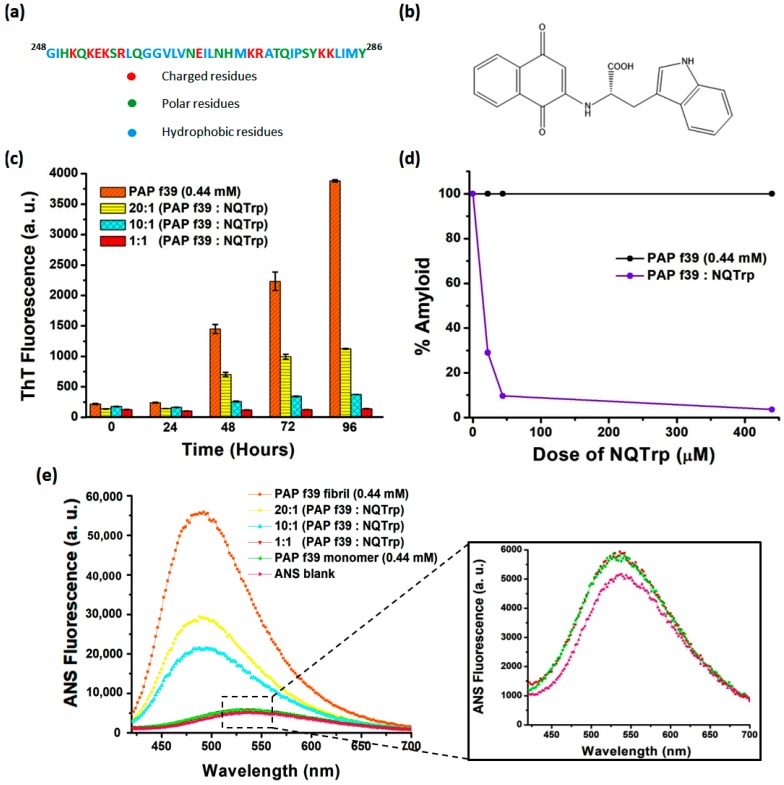
(**a**) Amino acid residues of the PAP f39 peptide fragment. (**b**) Molecular structure of NQTrp. (**c**) ThT fluorescence assay showing the inhibition of PAP f39 peptide aggregation in the absence and presence of NQTrp. (**d**) Plot showing % amyloid remaining in the mixture after the inhibition assay. (**e**) ANS fluorescence assay showing the dose-dependent inhibition of PAP f39 aggregation by NQTrp; inset shows the overlapping ANS emission spectra of PAP f39 treated with an equimolar ratio of NQTrp and the PAP f39 monomer along with the ANS blank.

Acquired immunodeficiency syndrome (AIDS) caused by the human immunodeficiency virus (HIV-1) is one of the top ten pandemics and has so far led to the deaths of >35 million people (http://www.who.int/gho/hiv/en/). Being a major contagious disease, the sexual transmission of the virus accounts for ~80% of total HIV infection [1,2]. Recently, it has been reported that a 39 amino acid protease cleavable peptide fragment of prostatic acid phosphatase (PAP_248–286_, henceforth PAP f39, Figure 1a) found in human semen forms amyloid fibrils termed SEVI (semen-derived enhancer of viral infection) and plays a crucial role in enhancing HIV infection by ~10^5^ fold [3]. Experimental data have shown that PAP f39 fibrils are highly cationic in nature [4], which facilitates the formation of an electrostatic bridge between the negatively charged cell and viral membrane, leading to increased viral attachment and fusion to target cells. This mechanism of enhancement of viral infection by PAP f39 is valid only in its amyloidogenic state and not by the freshly dissolved monomeric peptide [5,6,7,8]. Therefore, inhibiting amyloid aggregation of PAP f39 appears to be an attractive way to slow down HIV transmission.

Several approaches have been attempted in the past decade to minimize the viral infection-enhancing activity of semen amyloids for lowering the sexual transmission of HIV, some of which include (i) inhibiting the proteolytic cleavage of PAP to form PAP f39; (ii) inhibiting the conversion of PAP f39 monomers to infection-promoting amyloids; (iii) remodeling the existing fibrils to quantitatively reduce amyloid load; and (iv) neutralizing the charged surface of the fibrils and in turn disrupting the ability of the fibrils to mediate interaction between viruses and cells [4,9]. Agents such as small molecules e.g., epigallocatechin-3-gallate, brazilin, gallic acid, surfen, BTA-EG6, ADS-J1, and CLR01 [10,11,12,13,14,15,16]; peptide-based e.g., D3 (d-enantiomeric cationic peptide) and WW61 [17,18]; metal ions e.g., Cu^2+^ and Zn^2+^ [19]; polymers e.g., polyanions (heparin, dextran sulfate) [20,21] and BTA oligomers [9]; nanoparticles e.g., BTA-containing nanoparticles and hydrophobic nanoparticles [22,23] have been utilized to reduce SEVI and/or HIV transmission. Among these strategies, inhibiting PAP f39 aggregation seems more reliable since the inhibition of amyloids at early stages may render effective prevention, similar to the amyloids implicated in Alzheimer’s or Parkinson’s diseases [24,25,26].

Previously, we have demonstrated that a naphthoquinone tryptophan hybrid (NQTrp, Figure 1b) effectively inhibited the formation of a wide range of amyloids dominated by hydrophobic patches including those formed by Aβ, Tau, and α-synuclein implicated in neurodegenerative disorders in vitro, and also ameliorated their symptoms in transgenic Drosophila fly models [27,28,29]. Since NQTrp is an established generic inhibitor of amyloid aggregation [30], in the present study, we wished to determine whether it could inhibit the semen amyloids of the “hydrophilic” PAP f39 peptide. To this end, using in vitro methods, we examined the ability of NQTrp to inhibit the formation of PAP f39 fibrillar aggregates and in silico approaches to delineate the plausible mechanism of inhibition and to visualize the predicted binding sites of NQTrp with the PAP f39 monomer. Insights obtained from this work may provide a basis for designing targeted inhibitors for SEVI and other semen amyloids.

## 2. Results and Discussion

In the present work, we wanted to test whether NQTrp could inhibit the human semen amyloid. To that end, a monomeric peptide was allowed to aggregate and form amyloid fibrils either in the absence or presence of increasing concentrations of NQTrp (20:1, 10:1, and 1:1 of PAP f39:NQTrp, respectively) and the rate of amyloid aggregation was monitored using the thioflavin T (ThT) assay, 8-anilino-1-naphthalenesulfonic acid (ANS) assay, Congo red birefringence assay, and transmission electron microscopy analysis.

### 2.1. NQTrp Demonstrates Dose-Dependent Inhibition toward PAP f39 Amyloid Aggregation

ThT is a benzothiazole based amyloid reporter dye, which is barely fluorescent when free in solution, but shows enhancement in fluorescence intensity upon binding cross β-sheet rich structures as observed in amyloid fibrils [31]. The ThT fluorescence assay revealed that PAP f39 (0.44 mM) peptide monomers in the absence of NQTrp formed amyloid aggregates after 96 h under optimized aggregation conditions. This was evidenced from the drastic enhancement of the ThT fluorescence emission intensity observed (Figure 1c). The ThT fluorescence curve resulted in a sigmoidal pattern with a prominent lag phase till 24 h corresponding to the nucleation stage, and was later followed by the exponential increase and plateau fluorescence representing oligomerization and fibril maturation, respectively. These aggregation kinetics appeared similar to the nucleation-dependent polymerization model for amyloidogenic proteins [32] as observed previously for PAP f39 aggregation [7,33,34,35].

In a parallel aggregation assay, PAP f39 (0.44 mM) peptide monomers were allowed to aggregate in the presence of different doses of NQTrp (0.022 mM, 0.044 mM, and 0.44 mM) from time t = 0 h. PAP f39 aggregation was monitored by ThT fluorescence, where aggregation was found to be significantly reduced by NQTrp treatment in a dose-dependent manner (Figure 1c). Since there was a prolonged lag phase in the NQTrp treated samples, we hypothesized that NQTrp might have interacted with the PAP f39 peptides at the early nucleation stage to slow-down the aggregation kinetics. To rule out the likelihood of ThT fluorescence quenching by NQTrp, ThT (50 μM) and NQTrp (0.022 mM, 0.044 mM, and 0.44 mM) were co-incubated in the buffer in the absence of PAP f39 monomers. Emission of ThT fluorescence indicated that NQTrp had a very minimal quenching effect, hence did not significantly alter the outcomes of the inhibition assay (Figure S1).

ThT fluorescence can be used as a quantifiable tool to compare amyloid content between independent samples provided all other aggregation reaction parameters are kept unchanged [36]. To measure the inhibition of PAP f39 aggregation by NQTrp, a plot of % amyloid vs. NQTrp dose was generated. As shown in Figure 1d, a low concentration ratio of 20:1 (PAP f39:NQTrp) exhibited ~70% amyloid inhibition, and almost complete inhibition of PAP f39 aggregation was observed at an equimolar ratio.

### 2.2. NQTrp Retains the Native Conformation of the PAP f39 Monomers

To corroborate the findings obtained from the ThT assay, an ANS based fluorescence assay was performed. Similar to ThT, ANS is an extrinsic fluorescence probe, which when free in aqueous solutions is weakly fluorescent with λ_ex_ = 380 nm and λ_em_ = 535 nm [37]. However, the dye becomes extremely fluorescent when bound to hydrophobic patches of amyloid structures. Additionally, this binding causes a blue wavelength shift in the emission spectrum (λ_em_ = 460–490 nm) with a higher quantum yield when compared to free dye in solution [38]. Figure 1e shows that when the monomeric solution of PAP f39 was mixed with ANS, the emission peak was visualized at 535 nm with a basal level fluorescence similar to the ANS blank (Figure 1e, inset), which confirmed that the peptide was in native conformation. In contrast, when ANS was incubated with pre-formed PAP f39 aggregates in the absence of NQTrp (control), a significant blue shift of the emission peak (λ_em_ = 490) with several-fold enhancement of fluorescence was observed, which validated the presence of amyloid structures.

Next, we analyzed the spectra of PAP f39 aggregated in the presence of various doses of NQTrp, incubated with ANS post-aggregation reaction. The intensity of the emission spectra at λ_em_ = 490 was found to decrease with increasing concentrations of NQTrp (Figure 1e). It is noteworthy that at a 1:1 molar ratio (PAP f39:NQTrp), the ANS emission spectra post-aggregation overlapped that of the monomeric PAP f39 incubated with ANS (Figure 1e, inset). This result suggests that NQTrp could stabilize the native conformation of PAP f39 and effectively reduced the conversion of monomers to amyloid assemblies. The role of non-covalent interactions such as hydrogen bonding, π–π stacking and other hydrophobic interactions in facilitating and stabilizing the core of amyloid assemblies is well documented [39,40,41]. We have reported that NQTrp hybrids (i.e., NQTrp and Cl-NQTrp) form hydrogen bonds and π–π stacking with the key residues of Aβ and PHF6 peptides to inhibit their respective in vitro aggregation [27,29]. Since NQTrp preserved the native monomeric conformation of PAP f39, it is plausible that interaction between NQTrp and the amino acid residues of PAP f39 could have occurred at the early stages of aggregation through non-covalent contacts.

### 2.3. Congo Red Birefringence Revealed That NQTrp Minimize PAP f39 Amyloid Deposits

**Figure 2:**
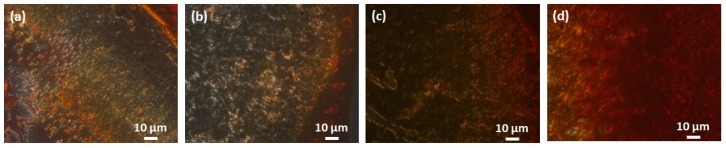
Representative Congo red birefringence images showing a decrease in amyloid load after incubation with various doses of NQTrp: (**a**) Control, i.e., aggregates of PAP f39 (440 μM) in the absence of NQTrp; Treatment, i.e., molar ratio of PAP f39:NQTrp: (**b**) 20:1, (**c**) 10:1, (**d**) 1:1.

To further validate whether NQTrp reduces PAP f39 amyloid aggregation, a Congo red birefringence assay was performed (Figure 2a–d). Congo red is an amyloid staining dye, which binds with amyloid fibrils and produces a characteristic apple-green birefringence under cross-polarized light [42]. Control PAP f39, i.e., in the absence of NQTrp, rendered a strong birefringence under cross-polarized light indicating self-assembly into amyloid fibrils (Figure 2a). The Congo red birefringence signal gradually decreased in samples incubated with various doses of NQTrp (Figure 2b,c). At the highest molar ratio of PAP f39:NQTrp (1:1), almost no apple-green birefringence was observed, suggesting a significant reduction in the amyloid content (Figure 2d).

### 2.4. Morphology of the Inhibited PAP f39 Assemblies

**Figure 3:**
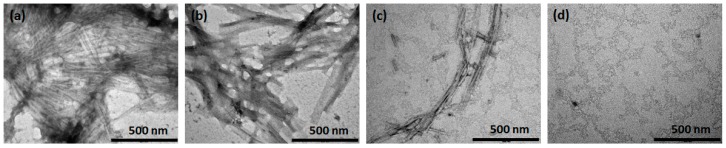
Representative TEM images showing the inhibition of fibril formation in the absence or presence of various molar ratio of PAP f39:NQTrp. (**a**) Control, i.e., aggregates of PAP f39 (440 μM) in the absence of NQTrp, (**b**) 20:1, (**c**) 10:1, and (**d**) 1:1.

Transmission electron microscopy (TEM) analysis of the PAP f39 fibrils formed in the absence and presence of NQTrp at molar ratios 20:1, 10:1, and 1:1 (PAP f39:NQTrp) was performed, and the representative images are shown in Figure 3a–d. In the absence of NQTrp, i.e., the PAP f39 control, the peptide fibrils appeared mature, long, and dense (Figure 3a). In contrast, we observed fibrils with broken morphology and a prominent decrease in the fibril density in a dose-dependent manner when treated with NQTrp (Figure 3b,c). At an equimolar concentration of 1:1 (PAP f39:NQTrp), the density of the fibrils was significantly reduced, and no elongated fibrillar structures were visualized (Figure 3d). This data substantiate the insights obtained from the ThT, ANS, and birefringence assays, all of which together show that NQTrp efficiently inhibited the formation of PAP f39 amyloids.

### 2.5. Modulating PAP f39 Aggregation by NQTrp Renders Non-Toxic Intermediates

**Figure 4:**
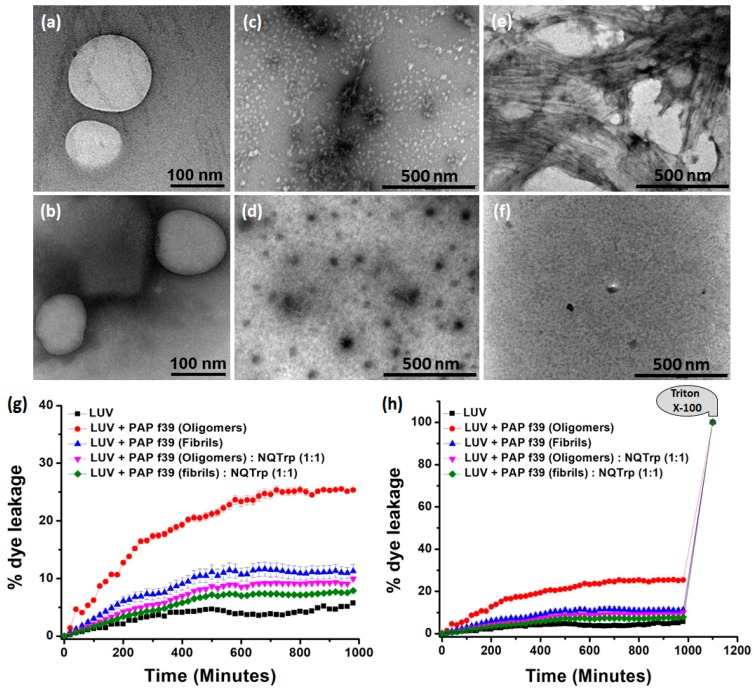
(**a**,**b**) TEM images of the large unilamellar vesicles (LUVs) (stock concentration 2 mM) in HEPES buffer (50 mM), pH 7.4. Images were taken immediately after the preparation of the LUVs. TEM images of PAP f39 oligomers in the (**c**) absence or (**d**) presence of NQTrp; TEM images of PAP f39 fibrils in the (**e**) absence or (**f**) presence of NQTrp used for LUV studies. (**g**) Plot showing % dye leakage from LUVs in the absence and presence of different PAP f39 preparations. (**h**) Plot showing % dye leakage from LUVs in the absence and presence of different PAP f39 preparations, with reference to the Triton X-100 treatment.

Oligomers of amyloidogenic proteins/peptides are considered to be the toxic species when compared to fibrils since they can rupture the cell membrane resulting in cell death [43,44]. Large unilamellar vesicles (LUVs) are commonly used as a model to mimic the cell membrane, and the disruption of these artificial vesicles is well established as a proxy for cytotoxicity [29,45,46]. To examine whether the oligomers resulting from PAP f39 aggregation can disrupt the LUV membrane, and whether NQTrp modulates it, a vesicle dye leakage assay was performed using carboxyfluorescein entrapped LUVs. Prior to the leakage assay, the formation and integrity of the LUVs were confirmed by TEM analysis (Figure 4a,b). The LUVs were found to be ~100–200 nm in diameter and had a uniform spherical morphology. Next, PAP f39 samples were prepared by allowing the monomers to aggregate, resulting in the formation of oligomers and subsequently fibrils, as described in Section 3.3. Since PAP f39 forms mature fibrils at ~96 h (Figure 1c), samples retrieved at ~48 h were oligomeric species as verified by TEM (Figure 4c). Samples containing untreated LUVs, i.e., without PAP f39 oligomers or fibrils, were used as the negative control and LUVs incubated with PAP f39 oligomer or fibrillar preparations (Figure 4c,e) in the absence of NQTrp were used as the positive control. Test sample preparations containing oligomers treated with NQTrp (PAP f39:NQTrp; 1:1) (Figure 4d) or fibrils treated with NQTrp (PAP f39:NQTrp; 1:1) (Figure 4f) were added separately to the LUVs, maintaining a lipid to PAP f39 molar ratio of 1:20. Triton X-100 (non-ionic detergent) was used as a reference for complete dye release from the LUVs, and the final fluorescence readout was measured according to the below equation [47]:

$$\%\;\mathrm{dye}\;\mathrm{leakage}=\frac{{\mathrm{Fluorescence}}_{\mathrm{observed}}-{\mathrm{Fluorescence}}_{\mathrm{initial}}}{{\mathrm{Fluorescence}}_{\mathrm{total}}-{\mathrm{Fluorescence}}_{\mathrm{initial}}}\times 100$$

Natural dye leakage from LUVs, i.e., background fluorescence, was minimal (5%) and remained relatively constant after 400 min. LUVs treated with PAP f39 oligomers and fibrils in the absence of NQTrp resulted in a marked increase of 26% and 12% dye release, respectively (Figure 4g,h). This result indicated that the oligomers significantly interacted with the vesicular membrane and ruptured the LUVs causing dye leakage, suggesting that PAP f39 oligomers are more toxic than the fibrillar species. In contrast, upon treatment of PAP f39 oligomers and fibrils with NQTrp, the % dye leakage was reduced to 9% and 7%, respectively (Figure 4g,h) indicating that NQTrp treatment gave rise to non-toxic intermediates and reduced the toxicity of higher order PAP f39 assemblies.

### 2.6. Interaction of NQTrp with PAP f39 Is Spontaneous and Involves Non-Covalent Contacts with Polar and Charged Amino Acid Residues

**Figure 5:**
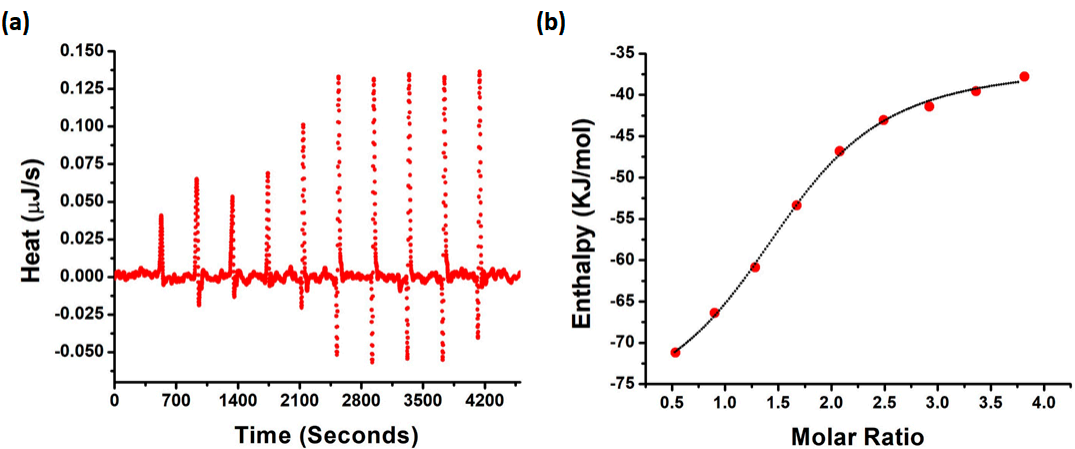
(**a**) Raw data of the heat pattern during PAP f39-NQTrp binding. (**b**) Curve showing the enthalpy changes with increasing PAP f39 to NQTrp mole fraction. Data were fitted using an independent binding model.

To evaluate the thermodynamic properties of NQTrp binding with PAP f39, we performed isothermal titration calorimetry (ITC) measurements. A fresh monomeric preparation of PAP f39 (350 μM) was titrated into a cell containing NQTrp (30 μM) to measure the corrected heat and the enthalpy value. Results of the titration profile and the thermodynamic values were calculated and are displayed in Figure 5a,b and Table 1. We found that the titration of PAP f39 to NQTrp resulted in exothermal peaks, whereas the titration of PAP f39 to a blank (i.e., PBS with no NQTrp) resulted in endothermal peaks. Gibbs free energy (ΔG) was calculated from the enthalpy (ΔH) and entropy (ΔS) values. ΔG was found to be negative (−30.97 KJ/mol), signifying that the binding of NQTrp to PAP f39 is spontaneous. Additionally, negative values for both TΔS (−11.48 kJ/mol) and ΔH (−42.51 kJ/mol) were observed at 37 °C. The absolute value of ΔH was larger than TΔS, suggesting that the interaction between NQTrp and PAP f39 is an enthalpy-driven process. It has been reported that the enthalpy-favored binding occurs through hydrogen bonding and electrostatic interactions, whereas entropy-favored binding occurs through hydrophobic contacts [48,49]. Therefore, our results indicate that the binding of NQTrp to PAP f39 was preferentially due to hydrogen bonding and electrostatic interactions. Additionally, the binding constant (K_d_) value of 5.94 μM supported the strong affinitive binding of NQTrp to PAP f39 monomers. The stoichiometric ratio (n = 1.5) indicates that one NQTrp molecule interacted with more than one PAP f39 monomer, which in turn points that NQTrp binds to PAP f39 at early stages of aggregation.

**Figure 6:**
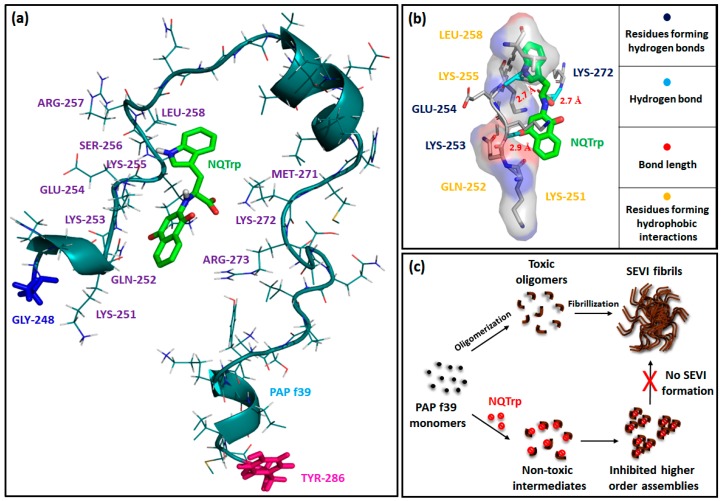
(**a**) Best docking conformer of PAP f39 peptide monomer with NQTrp; blue stick and pink stick: N-terminal glycine and C-terminal tyrosine of the PAP f39 monomer, respectively. Green stick: NQTrp. (**b**) Post-docking analysis and visualization of the interacting amino acid residues: NQTrp predominantly forms non-covalent contacts with polar and charged residues of PAP f39. (**c**) Cartoon showing plausible mechanism of PAP f39 aggregation inhibition by NQTrp; on pathway (absence of NQTrp): leads to oligomerization, fibrillization, and SEVI formation and off pathway (presence of NQTrp): leads to non-toxic intermediates and the inhibition of higher-order assemblies.

Furthermore, a molecular docking study was performed to determine the putative PAP f39 amino acid residues interacting with NQTrp and to obtain an atomistic insight on the binding mechanism underlying aggregation inhibition. The results of the docking analysis are summarized in Figure 6a,b and Figure S2 and Table S1. The docking of NQTrp with the PAP f39 monomer generated ten possible binding conformations. In the best-docked conformer (Figure 6a), NQTrp interacts predominantly with two regions of the peptide: Region 1—LYS 251 to LEU 258, and Region 2—MET 271 to ARG 273 with a strong binding energy of −7.7 kcal/mol. It is important to note that these binding regions are enriched with charged and polar residues such as LYS, ARG, SER, GLU, and GLN. This finding is in line with the NMR study, which showed that EGCG interacts with the LYS 251 to ARG 257 and ASN 269 to ILE 277 regions in the PAP f39 peptide, leading to amyloid disruption [50]. Since the binding regions consist of several charged residues, we were interested in exploring the nature of the interactions between PAP f39 and NQTrp. To this end, post-docking analysis was performed to visualize non-covalent contacts, if any.

Interestingly, this analysis indicated that NQTrp forms hydrogen bonds with LYS 253, GLU 254, and LYS 272 and facilitates hydrophobic contacts with LYS 251, GLN 252, LYS 255, and LEU 258 (Figure 6b and Figure S3, Table 2). Previous studies have advocated a crucial role of LYS residues for modulating PAP f39 aggregation by small molecules such as EGCG and CLR01 [16,50]. Furthermore, hydrophobic contacts were found to be the major players for the brazilin arbitrated inhibition of PAP f39 aggregation [11]. Harmonized with these conclusions, our docking data demonstrated that NQTrp predominantly interacts with the LYS residues and forms non-covalent complexes that are likely mediated by both hydrogen bonds and hydrophobic contacts.

Based on the outcomes of the biophysical assays, vesicle leakage assay, ITC, and molecular docking, we postulated the following as the mechanism of NQTrp mediated inhibition of PAP f39 aggregation: NQTrp interferes with PAP f39 at the early nucleation stage, and alters the molecular conformation of the peptide to render non-toxic intermediates. The interaction between PAP f39-NQTrp is spontaneous and mediated via non-covalent contacts with charged and polar amino acid residues. This complexation lowers the rate of aggregation kinetics and consequently inhibits elongation and propagation of higher order aggregates, thus abrogating SEVI formation (Figure 6c).

## 3. Materials and Methods

### 3.1. Materials

All chemicals and reagents were of analytical grade. Unless otherwise stated, all chemicals were obtained from Sigma-Aldrich (Rehovot, Israel). Synthetic PAP f39 was purchased from GL Biochem (Shanghai, China).

### 3.2. Stock Preparation

A stock of 200 mL phosphate buffer saline (PBS, 1.6 g of NaCl, 0.04 g of KCl, 0.288 g of Na_2_HPO_4_, 0.048 g of KH_2_PO_4_), pH 7.3 was prepared and filtered through a 0.22 µm filter (Millex-GV, Merck Millipore, MA, USA). Lyophilized PAP f39 peptide was pretreated with HFIP for 10 min to ensure the monomeric form, and subsequently, the solvent was evaporated using a SpeedVac. A stock volume of 2 mg/mL PAP f39 was prepared by dissolving the resulting thin film of the peptide in PBS and sonicating for 5 min. A 100 mM stock solution of NQTrp was prepared in DMSO. The stock solution was diluted in PBS to a working concentration of 5 mM. A stock solution of Thioflavin T (4 mM) was prepared in PBS and filtered using a 0.22 µm syringe filter.

### 3.3. ThT Fluorescence-Based PAP f39 Aggregation and Inhibition Assay

PAP f39 (2 mg/mL) was allowed to aggregate in the absence or presence of NQTrp at various molar ratios (PAP f39:NQTrp—20:1, 10:1 and 1:1) in PBS. The reaction mixtures were incubated at 37 °C with continuous orbital shaking (1200 rpm) for 96 h. An aliquot of 10 µL was withdrawn from each reaction mixture at a regular interval of 12 h and frozen at −20 °C. At the end of 96 h, all the samples were thawed to room temperature, and ThT was added to a final concentration of 50 µM and incubated in the dark for 2 h at 37 °C. Samples were transferred to a 384-well flat black plate (Corning) and the ThT fluorescence intensity (λ_ex_ = 440 nm λ_em_ = 480 nm) was measured using a microplate reader (Infinite M200, Tecan, Switzerland). All measurements were performed in triplicate, and the assay was repeated three times to ensure reproducibility. Error bars in the figure represent standard error.

### 3.4. 8-Anilinonaphthalene-1-Sulfonic Acid (ANS) Binding Assay

PAP f39 samples (10 μL) aggregated in the absence or presence of different doses of NQTrp were mixed with an equimolar ratio of ANS and incubated in the dark for 2 h at room temperature. Samples were transferred to a 384-well flat black plate, and the ANS fluorescence intensity was recorded with λ_ex_ = 380 nm and λ_em_ between 420 nm and 700 nm. All measurements were performed in triplicate.

### 3.5. Congo Red Birefringence Assay

Congo red powder was dissolved in 80% aqueous ethanol to prepare a saturated stock solution. PAP f39 samples (5 μL) aggregated in the absence or presence of different doses of NQTrp were mixed with 5 μL of saturated Congo red solution. The suspension was air dried on a glass microscope slide and kept in a desiccator before birefringence analysis. Specimens were viewed at 60X magnification with a Nikon Eclipse TI polarizing microscope (Tokyo, Japan) Digitized images were obtained using a Nikon DS Ri1 digital camera (Tokyo, Japan).

### 3.6. Transmission Electron Microscopy

Samples of 10 µL were drop casted onto 400 mesh carbon-coated copper grids (Electron Microscopy Sciences (EMS), Hatfield, PA, USA) and allowed to adhere for 2 min. Excess fluid was removed, and the grids were negatively stained by using 2% uranyl acetate for 2 min. Finally, the excess fluid was removed, and the samples were viewed by a JEM-1400 TEM (JEOL, Tokyo, Japan), operated at 80 kV.

### 3.7. Vesicle Dye Leakage Assay

Vesicles were prepared as described previously [51,52]. Briefly, large unilamellar vesicles (LUVs) were prepared using three different lipids, DMPC, cholesterol, and GM1 with 68:30:2 molar ratios in 20 mM MOPS buffer of pH 7.2. All lipids were taken in a clean glass vessel and solubilized to make 1 mM stock solution in chloroform and methanol (2:1), and the solvents were evaporated under vacuum. The lipid films were hydrated with 620 µL of carboxyfluorescein solution (100 µM) in 20 mM MOPS buffer and immediately vortexed vigorously for 40 min to emulsify the lipid mixtures. Then, the lipid solution was dipped into liquid nitrogen for instant cooling, and after 2 min, the frozen solution was dipped into a water bath at 50–60 °C for thawing. These steps of freeze-thaw were repeated five times, and excess dye was removed by ultracentrifugation at 20,000 rpm. The supernatant was discarded, and the lipid pellet was re-hydrated with 20 mM MOPS. This step was repeated two more times, and the final lipid pellet was collected, followed by the addition of 620 µL of MOPS buffer and vortexed to obtain a homogenous suspension of 1 mM of dye-loaded LUVs. The PAP f39 oligomers and fibrils were incubated with the LUVs, and the dye leakage study was performed in triplicate on a microplate reader.

### 3.8. Isothermal Titration Calorimetry

Fresh monomeric PAP f39 (350 μM) was dissolved in 40 mM PBS, and NQTrp was diluted in PBS to a working concentration of 30 μM. Both solutions were separately incubated for 15 min at 37 °C before the ITC measurements. A sample of 300 µL NQTrp was inserted into the Nano ITC low volume cell (TA Instruments, Newcastle, DE, USA) and the titrating syringe was filled with 50 μL PAP f39 solution. The system was allowed to reach a stable temperature of 37 °C along 2000 s and collected baseline for 500 s. Subsequently, PAP f39 was titrated to the NQTrp solution or PBS as a control. Titration was carried out in 5 μL aliquots and allowed to equilibrate for 400 s before the next drop, along ten drops, of total 47.5 μL (first drop was half volume). The resulted isotherm was analyzed using Nanoanalyze software using an independent interaction model. Baseline correction was performed by titrating PAP f39 to the PBS blank.

### 3.9. Molecular Docking

NMR structure of the PAP f39 peptide was retrieved from the Protein Data Bank (PDB ID: 2L3H) [53], and the 3D structure of NQTrp (CID: 56605052) was obtained from the PubChem database. The computational docking study of PAP f39 and NQTrp was performed using AutoDock 4.2 software (v1.5.6,) [54]. Water molecules and ions were removed from the initial peptide structure. Polar hydrogen atoms were added, and the Kollman united atomic partial charges were assigned to the peptide. The default search function in the Lamarckian genetic algorithm was used for the docking analysis. Docking of the ligand was performed on the whole peptide sequence. The grid maps representing the peptide were calculated using the auto grid, and the grid size was set to 65 × 60 × 60 points along the *X*, *Y*, and *Z* axes, respectively, with a grid spacing of 0.425 Ǻ. Three independent docking runs were carried out for the system. LigPlot+ [55] and PyMOL (https://pymol.org/2/) were used for the visualization and analysis of the docked conformations.

## 4. Conclusions

Semen amyloids resulting from PAP f39 aggregation are implicated in enhancing the sexual HIV transmission in AIDS. Therefore, modulating PAP f39 aggregation might be an effective treatment strategy. In the present work, we have used in vitro and in silico techniques to portray the inhibitory effect of NQTrp toward PAP f39 self-assembly. We found that NQTrp has strong affinitive binding sites in PAP f39, which likely facilitate its association with the peptide at an early nucleation stage through non-covalent contacts. This results in the formation of non-toxic intermediates and eventually inhibits the progression of higher order aggregates. Taken together, our findings underscore the inhibitory capacity of NQTrp toward PAP f39 amyloid formation and project NQTrp as a potential scaffold for the design of novel small molecules that target semen amyloids.

## Figures and Tables

**Table 1:** Thermodynamic properties of NQTrp and PAP f39 binding as determined by the ITC measurements.

Parameter	Value	Standard Deviation
K_d_ (μM)	5.94	1.26
n	1.502	0.060
ΔH (kJ/mol)	−42.51	2.57
ΔS (J/mol·K)	−37.02	NA

**Table 2:** Summary of docking studies showing the NQTrp association with PAP f39.

Hydrogen Bonds		Hydrophobic Contacts	
Interacting Atom of Amino Acid Residues	Interacting Atoms of NQTrp	Residues	Region of NQTrp
N1 of Lys_253_	O4	Lys_251_	Naphthoquinone ring
O1 of Glu_254_	N2	Gln_252_	Naphthoquinone ring
N2 of Lys_272_	O2	Lys_255_	Indole ring
		Leu_258_	Indole ring

## Supplementary Materials

The following are available online, Figure S1: NQTrp shows minimal quenching of ThT fluorescence. ThT (50 μM) was incubated with various concentrations of NQTrp. ThT fluorescence emission was measured at 480 nm by exciting the dye at 440 nm; Figure S2: Various docking conformations of the PAP f39 monomer and NQTrp; Figure S3: Docking study: Association of NQTrp with PAP f39; Table S1: Post-docking analysis shows the interaction profile of NQTrp with the PAP f39 monomer corresponding to the docked conformers in Figure S2.

## References

[1] Gray R.H., Wawer M.J., Brookmeyer R., Sewankambo N.K., Serwadda D., Wabwire-Mangen F., Lutalo T., Li X., vanCott T., Quinn T.C. (2001). Probability of HIV-1 transmission per coital act in monogamous, heterosexual, HIV-1-discordant couples in Rakai, Uganda. Lancet.

[2] Pilcher C.D., Tien H.C., Eron J.J.J., Vernazza P.L., Leu S.-Y., Stewart P.W., Goh L.-E., Cohen M.S. (2004). Brief but efficient: Acute HIV infection and the sexual transmission of HIV. J. Infect. Dis..

[3] Munch J., Rucker E., Standker L., Adermann K., Goffinet C., Schindler M., Wildum S., Chinnadurai R., Rajan D., Specht A. (2007). Semen-derived amyloid fibrils drastically enhance HIV infection. Cell.

[4] Röcker A., Roan N.R., Yadav J.K., Fändrich M., Münch J. (2018). Structure, function and antagonism of semen amyloids. Chem. Commun..

[5] Kim K.-A., Yolamanova M., Zirafi O., Roan N.R., Staendker L., Forssmann W.-G., Burgener A., Dejucq-Rainsford N., Hahn B.H., Shaw G.M. (2010). Semen-mediated enhancement of HIV infection is donor-dependent and correlates with the levels of SEVI. Retrovirology.

[6] Castellano L.M., Shorter J. (2012). The Surprising Role of Amyloid Fibrils in HIV Infection. Biology.

[7] Olsen J.S., DiMaio J.T.M., Doran T.M., Brown C., Nilsson B.L., Dewhurst S. (2012). Seminal plasma accelerates semen-derived enhancer of viral infection (SEVI) fibril formation by the prostatic acid phosphatase (PAP248-286) peptide. J. Biol. Chem..

[8] Roan N.R., Greene W.C. (2007). A seminal finding for understanding HIV transmission. Cell.

[9] Capule C.C., Brown C., Olsen J.S., Dewhurst S., Yang J. (2012). Oligovalent amyloid-binding agents reduce SEVI-mediated enhancement of HIV-1 infection. J. Am. Chem. Soc..

[10] Hauber I., Hohenberg H., Holstermann B., Hunstein W., Hauber J. (2009). The main green tea polyphenol epigallocatechin-3-gallate counteracts semen-mediated enhancement of HIV infection. Proc. Natl. Acad. Sci. USA.

[11] Li M., Dong X., Liu Y., Sun Y. (2017). Brazilin Inhibits Prostatic Acidic Phosphatase Fibrillogenesis and Decreases its Cytotoxicity. Chem. Asian J..

[12] LoRicco J.G., Xu C.S., Neidleman J., Bergkvist M., Greene W.C., Roan N.R., Makhatadze G.I. (2016). Gallic Acid Is an Antagonist of Semen Amyloid Fibrils That Enhance HIV-1 Infection. J. Biol. Chem..

[13] Roan N.R., Sowinski S., Münch J., Kirchhoff F., Greene W.C. (2010). Aminoquinoline Surfen Inhibits the Action of SEVI (Semen-derived Enhancer of Viral Infection). J. Biol. Chem..

[14] Olsen J.S., Brown C., Capule C.C., Rubinshtein M., Doran T.M., Srivastava R.K., Feng C., Nilsson B.L., Yang J., Dewhurst S. (2010). Amyloid-binding Small Molecules Efficiently Block SEVI (Semen-derived Enhancer of Virus Infection)- and Semen-mediated Enhancement of HIV-1 Infection. J. Biol. Chem..

[15] Xun T., Li W., Chen J., Yu F., Xu W., Wang Q., Yu R., Li X., Zhou X., Lu L. (2015). ADS-J1 Inhibits Semen-Derived Amyloid Fibril Formation and Blocks Fibril-Mediated Enhancement of HIV-1 Infection. Antimicrob. Agents Chemother..

[16] Lump E., Castellano L.M., Meier C., Seeliger J., Erwin N., Sperlich B., Sturzel C.M., Usmani S., Hammond R.M., von Einem J. (2015). A molecular tweezer antagonizes seminal amyloids and HIV infection. Elife.

[17] Widera M., Klein A.N., Cinar Y., Funke S.A., Willbold D., Schaal H. (2014). The D-amino acid peptide D3 reduces amyloid fibril boosted HIV-1 infectivity. AIDS Res. Ther..

[18] Sievers S.A., Karanicolas J., Chang H.W., Zhao A., Jiang L., Zirafi O., Stevens J.T., Munch J., Baker D., Eisenberg D. (2011). Structure-based design of non-natural amino-acid inhibitors of amyloid fibril formation. Nature.

[19] Sheftic S.R., Snell J.M., Jha S., Alexandrescu A.T. (2012). Inhibition of semen-derived enhancer of virus infection (SEVI) fibrillogenesis by zinc and copper. Eur. Biophys. J..

[20] Arnold F., Schnell J., Zirafi O., Sturzel C., Meier C., Weil T., Standker L., Forssmann W.-G., Roan N.R., Greene W.C. (2012). Naturally occurring fragments from two distinct regions of the prostatic acid phosphatase form amyloidogenic enhancers of HIV infection. J. Virol..

[21] Roan N.R., Muller J.A., Liu H., Chu S., Arnold F., Sturzel C.M., Walther P., Dong M., Witkowska H.E., Kirchhoff F. (2011). Peptides released by physiological cleavage of semen coagulum proteins form amyloids that enhance HIV infection. Cell Host Microbe.

[22] Sheik D.A., Brooks L., Frantzen K., Dewhurst S., Yang J. (2015). Inhibition of the enhancement of infection of human immunodeficiency virus by semen-derived enhancer of virus infection using amyloid-targeting polymeric nanoparticles. ACS Nano.

[23] Sheik D.A., Chamberlain J.M., Brooks L., Clark M., Kim Y.H., Leriche G., Kubiak C.P., Dewhurst S., Yang J. (2017). Hydrophobic Nanoparticles Reduce the beta-Sheet Content of SEVI Amyloid Fibrils and Inhibit SEVI-Enhanced HIV Infectivity. Langmuir.

[24] Haj E., Losev Y., Guru KrishnaKumar V., Pichinuk E., Engel H., Raveh A., Gazit E., Segal D. (2018). Integrating in vitro and in silico approaches to evaluate the “dual functionality” of palmatine chloride in inhibiting and disassembling Tau-derived VQIVYK peptide fibrils. Biochim. Biophys. Acta.

[25] Habchi J., Chia S., Limbocker R., Mannini B., Ahn M., Perni M., Hansson O., Arosio P., Kumita J.R., Challa P.K. (2016). Systematic development of small molecules to inhibit specific microscopic steps of Aβ42 aggregation in Alzheimer’s disease. Proc. Natl. Acad. Sci. USA.

[26] Kurnik M., Sahin C., Andersen C.B., Lorenzen N., Giehm L., Mohammad-Beigi H., Jessen C.M., Pedersen J.S., Christiansen G., Petersen S.V. (2018). Potent alpha-Synuclein Aggregation Inhibitors, Identified by High-Throughput Screening, Mainly Target the Monomeric State. Cell Chem. Biol..

[27] Scherzer-Attali R., Pellarin R., Convertino M., Frydman-Marom A., Egoz-Matia N., Peled S., Levy-Sakin M., Shalev D.E., Caflisch A., Gazit E. (2010). Complete phenotypic recovery of an Alzheimer’s disease model by a quinone-tryptophan hybrid aggregation inhibitor. PLoS ONE.

[28] Frenkel-Pinter M., Tal S., Scherzer-Attali R., Abu-Hussien M., Alyagor I., Eisenbaum T., Gazit E., Segal D. (2016). Naphthoquinone-Tryptophan Hybrid Inhibits Aggregation of the Tau-Derived Peptide PHF6 and Reduces Neurotoxicity. J. Alzheimer’s Dis..

[29] KrishnaKumar V.G., Paul A., Gazit E., Segal D. (2018). Mechanistic insights into remodeled Tau-derived PHF6 peptide fibrils by Naphthoquinone-Tryptophan hybrids. Sci. Rep..

[30] Scherzer-Attali R., Shaltiel-Karyo R., Adalist Y.H., Segal D., Gazit E. (2012). Generic inhibition of amyloidogenic proteins by two naphthoquinone-tryptophan hybrid molecules. Proteins.

[31] Groenning M. (2009). Binding mode of Thioflavin T and other molecular probes in the context of amyloid fibrils-current status. J. Chem. Biol..

[32] Chatani E., Yamamoto N. (2017). Recent progress on understanding the mechanisms of amyloid nucleation. Biophys. Rev..

[33] French K.C., Makhatadze G.I. (2012). Core sequence of PAPf39 amyloid fibrils and mechanism of pH-dependent fibril formation: The role of monomer conformation. Biochemistry.

[34] Srivastava K.R., French K.C., Tzul F.O., Makhatadze G.I., Lapidus L.J. (2016). Intramolecular diffusion controls aggregation of the PAPf39 peptide. Biophys. Chem..

[35] Tan S., Lu L., Li L., Liu J., Oksov Y., Lu H., Jiang S., Liu S. (2013). Polyanionic candidate microbicides accelerate the formation of semen-derived amyloid fibrils to enhance HIV-1 infection. PLoS ONE.

[36] Xue C., Lin T.Y., Chang D., Guo Z. (2017). Thioflavin T as an amyloid dye: Fibril quantification, optimal concentration and effect on aggregation. R. Soc. Open Sci..

[37] Weber G., Laurence D.J. (1954). Fluorescent indicators of adsorption in aqueous solution and on the solid phase. Biochem. J..

[38] Hawe A., Sutter M., Jiskoot W. (2008). Extrinsic Fluorescent Dyes as Tools for Protein Characterization. Pharm. Res..

[39] Goto Y., Yagi H., Yamaguchi K., Chatani E., Ban T. (2008). Structure, formation and propagation of amyloid fibrils. Curr. Pharm. Des..

[40] Gazit E. (2002). A possible role for pi-stacking in the self-assembly of amyloid fibrils. FASEB J..

[41] KrishnaKumar V.G., Baweja L., Ralhan K., Gupta S. (2018). Carbamylation promotes amyloidogenesis and induces structural changes in Tau-core hexapeptide fibrils. Biochim. Biophys. Acta Gen. Subj..

[42] Westermark G.T., Johnson K.H., Westermark P. (1999). Staining methods for identification of amyloid in tissue. Methods Enzymol..

[43] Ward S.M., Himmelstein D.S., Lancia J.K., Binder L.I. (2012). Tau oligomers and tau toxicity in neurodegenerative disease. Biochem. Soc. Trans..

[44] Sengupta U., Nilson A.N., Kayed R. (2016). The Role of Amyloid-β Oligomers in Toxicity, Propagation, and Immunotherapy. EBioMedicine.

[45] Flach K., Hilbrich I., Schiffmann A., Gartner U., Kruger M., Leonhardt M., Waschipky H., Wick L., Arendt T., Holzer M. (2012). Tau oligomers impair artificial membrane integrity and cellular viability. J. Biol. Chem..

[46] Paul A., Nadimpally K.C., Mondal T., Thalluri K., Mandal B. (2015). Inhibition of Alzheimer’s amyloid-beta peptide aggregation and its disruption by a conformationally restricted alpha/beta hybrid peptide. Chem. Commun..

[47] McLaurin J., Chakrabartty A. (1996). Membrane disruption by Alzheimer beta-amyloid peptides mediated through specific binding to either phospholipids or gangliosides. Implications for neurotoxicity. J. Biol. Chem..

[48] Ross P.D., Subramanian S. (1981). Thermodynamics of protein association reactions: Forces contributing to stability. Biochemistry.

[49] Wang S.-H., Liu F.-F., Dong X.-Y., Sun Y. (2010). Thermodynamic analysis of the molecular interactions between amyloid beta-peptide 42 and (−)-epigallocatechin-3-gallate. J. Phys. Chem. B.

[50] Popovych N., Brender J.R., Soong R., Vivekanandan S., Hartman K., Basrur V., Macdonald P.M., Ramamoorthy A. (2012). Site specific interaction of the polyphenol EGCG with the SEVI amyloid precursor peptide PAP(248-286). J. Phys. Chem. B.

[51] Paul A., Kalita S., Kalita S., Sukumar P., Mandal B. (2017). Disaggregation of Amylin Aggregate by Novel Conformationally Restricted Aminobenzoic Acid containing α/β and α/γ Hybrid Peptidomimetics. Sci. Rep..

[52] Williams T.L., Day I.J., Serpell L.C. (2010). The effect of Alzheimer’s Abeta aggregation state on the permeation of biomimetic lipid vesicles. Langmuir.

[53] Nanga R.P.R., Brender J.R., Vivekanandan S., Popovych N., Ramamoorthy A. (2009). NMR structure in a membrane environment reveals putative amyloidogenic regions of the SEVI precursor peptide PAP(248-286). J. Am. Chem. Soc..

[54] Morris G.M., Huey R., Lindstrom W., Sanner M.F., Belew R.K., Goodsell D.S., Olson A.J. (2009). AutoDock4 and AutoDockTools4: Automated Docking with Selective Receptor Flexibility. J. Comput. Chem..

[55] Laskowski R.A., Swindells M.B. (2011). LigPlot+: Multiple ligand-protein interaction diagrams for drug discovery. J. Chem. Inf. Model..

