# Supplementary material for: Inhibitory Effect of Naphthoquinone-Tryptophan Hybrid towards Aggregation of PAP f39 Semen Amyloid
Source: Molecules. 2018 Dec 11;23(12):3279. doi: 10.3390/molecules23123279 (PMC6320874; doi:10.3390/molecules23123279)
Supplement: Supplementary file 1 [file molecules-23-03279-s001.pdf]

Supplementary data

Inhibitory effect of Naphthoquinone-Tryptophan hybrid towards aggregation of PAP f39 semen amyloid

V. Guru KrishnaKumar[1†], Satabdee Mohapatra[1†], Ashim Paul[1], Elad Arad[2], Raz Jelinek[2], Ehud Gazit[1] and Daniel Segal[1,3*]

[1]School of Molecular Microbiology & Biotechnology, Tel Aviv University, Tel Aviv 69978, Israel

[2]Department of Chemistry and Ilse Katz Institute (IKI) for Nanoscale Science and Technology, Ben Gurion University of the Negev, Beer Sheva 84105, Israel

[3]Sagol Interdisciplinary School of Neurosciences, Tel Aviv University, Tel Aviv 69978, Israel

[†] Contributed equally to the work

* Corresponding author; E-mail address: dsegal@post.tau.ac.il (Prof. Daniel Segal)

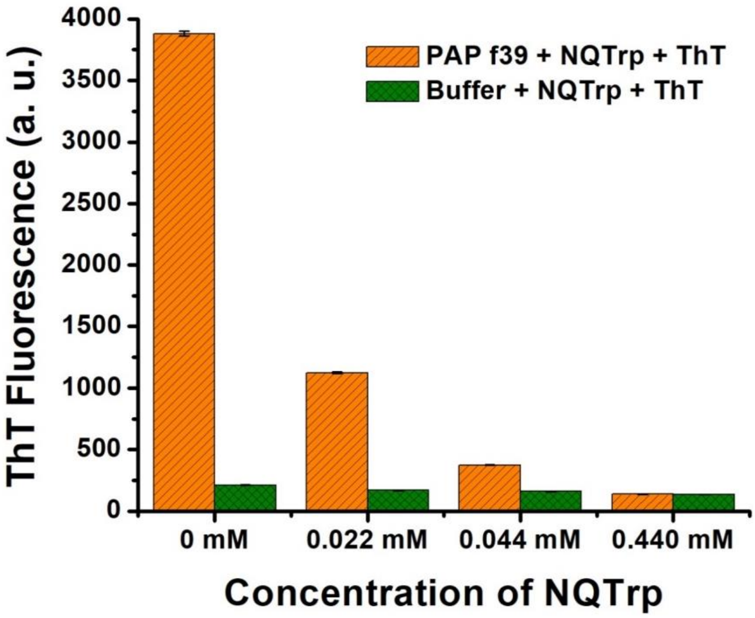

Figure S1. NQTrp shows minimal quenching of ThT fluorescence. ThT (50 μM) was incubated with various concentrations of NQTrp. ThT fluorescence emission was measured at 480 nm by exciting the dye at 440 nm.

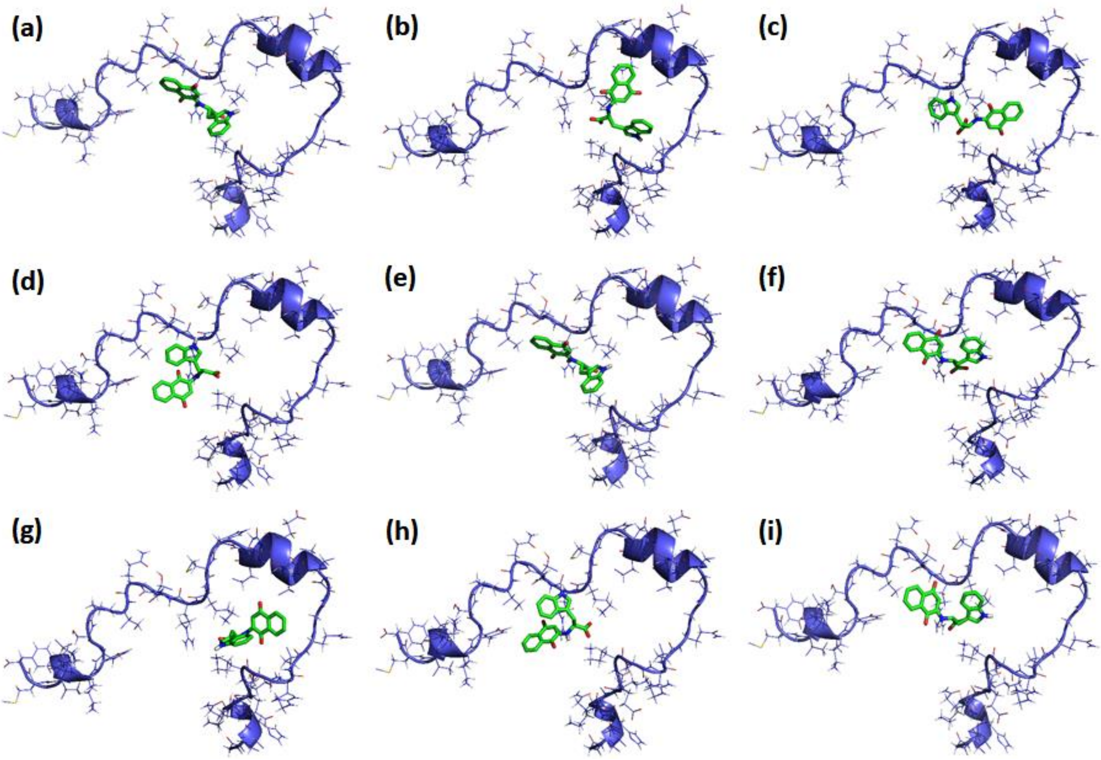

Figure S2. Various docking conformations of PAP f39 monomer and NQTrp.

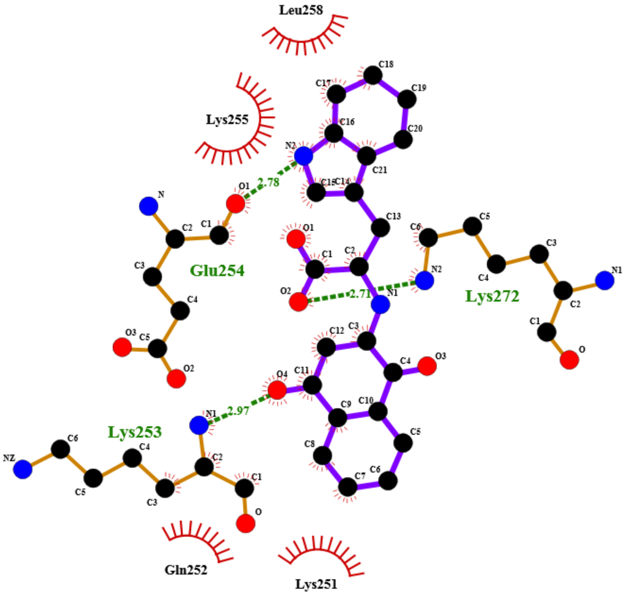

Figure S3. Docking study: Association of NQTrp with PAP f39.

Table S1. Post-docking analysis shows the interaction profile of NQTrp with PAP f39 monomer corresponding to the docked conformers in SI Fig. 2.

Notation	NQTrp association with $PAP_{248–286}$		Binding Energy
	Hydrogen bonds	Hydrophobic Interactions	(kcal/mol)
a	Lys_{272}	Lys_{255}, Arg_{273}, Ala_{274}, Thr_{275}, Ile_{277}	−6.99
b	Lys_{272}	Lys_{255}, Leu_{258}, Val_{264}, $Asn_{265,}$ Leu_{268}, Asn_{269}	−6.98
c	$Lys_{255,}$ Lys_{272}	Arg_{273}, Thr_{275}, Leu_{258}, Val_{264},	−6.94
d	Lys_{272}, Arg_{273}	Lys_{255}, Ala_{274}, Thr_{275}, Ile_{277}, Tyr_{280}	−6.78
e	Lys_{272}	Lys_{255}, Arg_{273}, Ala_{274}, Thr_{275}, Ile_{277},	−6.77
f	Lys_{272}	Lys_{255}, Arg_{273}, Thr_{275}, Ile_{277},	−6.69
g	–	Lys_{255}, Leu_{258}, $Gln_{259,}$ Val_{264}, Leu_{268}, Lys_{268}	−6.64
h	$Lys_{255,}$ Lys_{272}	Arg_{273}, Thr_{275}, Ile_{277}, Tyr_{280}	−6.49
i	Lys_{272}	Lys_{255}, Arg_{273}, Ala_{274}, Thr_{275}, Ile_{277},	−6.40
